# Relationship between physical performance and mild cognitive impairment in elderly hemodialysis patients is modified by the presence of diabetes: A multicenter cross-sectional study

**DOI:** 10.3389/fendo.2022.897728

**Published:** 2022-09-09

**Authors:** Yinjiao Zhao, Peiyu Song, Chan Zhu, Lingyun Zhang, Xiaoyu Chen, Hui Zhang, Peipei Han, Wei Ding, Jianying Niu, Junli Zhao, Xiang Shao, Liming Zhang, Chen Yu, Jia Xu, Chenghu Fang, Qi Guo

**Affiliations:** ^1^ Jiangwan Hospital of Hongkou District, Shanghai University of Medicine and Health Science Affiliated First Rehabilitation Hospital, Shanghai, China; ^2^ Department of Rehabilitation Medicine, Shanghai University of Medicine and Health Sciences, Shanghai, China; ^3^ Department of Nephrology, Shanghai Ninth People’s Hospital, Shanghai Jiaotong University School of Medicine, Shanghai, China; ^4^ Department of Nephrology, The Fifth People’s Hospital of Shanghai, Fudan University, Shanghai, China; ^5^ Department of Nephrology, Shanghai University of Medicine and Health Sciences Affiliated Zhoupu Hospital, Shanghai, China; ^6^ Department of Nephrology, Suzhou Kowloon Hospital, Shanghai Jiaotong University School of Medcine, Suzhou, China; ^7^ Department of Nephrology, Zhabei Central Hospital of Jingan District, Shanghai, China; ^8^ Department of Nephrology, Tongji Hospital, School of Medicine, Tongji University, Shanghai, China; ^9^ Department of Nephrology, Shanghai Pudong New Area People’s Hospital, Shanghai, China

**Keywords:** mild cognitive impairment, diabetes, physical performance, walking speed, hemodialysis

## Abstract

**Objective:**

The purpose of this study was to observe the relationship between physical performance and mild cognitive impairment (MCI) in the presence or absence of type 2 diabetes in elderly hemodialysis patients.

**Methods:**

In this multicenter cross-sectional study, 396 clinically stable and aged ≥60 years hemodialysis patients (255 men; mean age: 68.3 ± 5.9 years) were included from seven dialysis units in Shanghai, China. The Chinese version of the Modified Mini-Mental State Examination (MMSE) and the Instrumental Activities of Daily Living (IADL) scale were utilized to assess MCI. The performance-based assessments consisted of three physical tests, grip strength (GS), Timed Up and Go Test (TUGT), and 4-m walking test, which respectively represent muscle strength, mobility, and walking speed (WS). Logistic regression and multivariate linear regression were used for analysis.

**Results:**

Hemodialysis patients with diabetes had a high prevalence of MCI (20.6%). The odds ratio (OR) of MCI for the interacted items [(TUGT) * (diabetes) and (WS) * (diabetes)] was significant (p < 0.05). In diabetes patients, TUGT was positively associated with MCI, and WS was negatively associated with MCI after adjusting covariates [OR = 0.129; 95% confidence interval (CI) = 0.028–0.704, p = 0.021]. However, no significant association was found between physical performance and MCI in the non-diabetes hemodialysis patients (p > 0.05). Further analysis showed that TUGT was negatively associated with attention and calculation and language. WS was positively associated with recall and language in diabetic hemodialysis patients.

**Conclusions:**

Physical performance was associated with MCI in diabetic hemodialysis patients rather than the non-diabetes group. Whether increasing mobility or WS can positively influence MCI in individuals with type 2 diabetes requires further study.

## Introduction

Mild cognitive impairment (MCI) represents a transitional stage between normal age-related decline in cognitive function and dementia and is more prevalent in the elderly population and hemodialysis patients than the general population (1). As a therapeutic window, the latest guidelines show that MCI patients are still more likely to improve or maintain cognitive function (2). The decline in cognitive function is often influenced by many factors, such as age, education, vascular diseases, and chronic diseases (diabetes, hypertension) (3). Considering that chronic kidney disease (CKD) patients are usually accompanied by the protein-energy wasting and metabolic disorders that lead to impaired muscle mass and a decline in physical performance (4), the relationship between physical performance and MCI in elderly hemodialysis patients deserves further in-depth study.

Diabetes is considered to be a major cause of end-stage renal disease and appears to be increasing rapidly (5). Having prediabetes and diabetes was significantly associated with lower health-related quality of life relative to normal glucose tolerance (6). Data from a well-functioning population showed that compared with those without diabetes, those with diabetes exhibited lower performance on objective measures of lower-extremity function (7). The latest study showed a strong and significant correlation between 5-m gait speed and glycemia (8). Physical activity and sedentary behavior are associated with biomarkers of endothelial dysfunction, and the associations were stronger in (pre)diabetes than in normal glucose metabolism (9). In addition, a previous study has indicated a link of diabetes to an increased risk of MCI (10) and shown that the risk of incident MCI is higher in people with type 2 diabetes than that in those without diabetes (11). Pasquale et al. found a significant correlation between 5-m gait speed test and Montreal Cognitive Assessment (MoCA) score in frail diabetic older adults (12). Due to insufficient insulin secretion or insulin resistance, insulin-stimulated glucose uptake is markedly reduced in skeletal muscle and a hyperglycemic condition leads to endothelial and cerebral microvascular dysfunction (13–15), which may affect both physical performance and cognitive function. Previous and our studies have reported that poor physical performance is significantly associated with MCI in community-dwelling older adults (16–18). However, whether the presence of diabetes alters the relationship between physical performance and MCI is not yet known.

Therefore, this study aimed to explore the relationship between physical performance and MCI in elderly hemodialysis patients with and without diabetes. According to the above indications, we hypothesized that the presence of diabetes would lead to poorer physical performance and high prevalence of MCI, and different conditions may influence the association between physical performance and MCI. Moreover, it also investigated the association between physical performance [muscle strength, mobility, and walking speed (WS)] and specific cognitive functions in the presence or absence of type 2 diabetes to provide evidence for clinicians to effectively manage MCI in hemodialysis patients.

## Methods

### Study participants

The multicenter cross-sectional study recruited patients who underwent hemodialysis in seven dialysis units in Shanghai, China [ChiCTR1900027039] between July 2020 and April 2021. Hemodialysis is a process in which blood is drained outside the body through a circulatory line, exchanged through a dialyzer, and the purified blood is returned to the body. Vascular access modalities for hemodialysis included fistulas and catheters, and dialyzer models included F14, LOPS15, FX80, etc. Patients aged 60 years or older who were on hemodialysis for 4 h per session, three times a week, and for more than 3 months were included in the study. Participants with the following conditions were excluded: 1) unable to communicate with interviewers or grant informed consent; 2) unable to complete the physical performance test; 3) had a known diagnosis of dementia, psychiatric disorders, or other neurodegenerative diseases; and 4) no blood sample collection. Following these exclusions, the final analyzed population comprised 396 subjects (255 men, 141 women). All participants are required to complete an annual health screening and a detailed questionnaire on lifestyle and disease history. The study was approved by the Ethics Committee of the Shanghai University of Medicine and Health Sciences, and the methods were carried out in accordance with the principles of the Declaration of Helsinki. All participants were informed and signed consent prior to enrollment in the study.

### Baseline variables

Demographic characteristics (including age, gender, education level, registered residence, and marital status) and health behaviors (including smoking, drinking, and sleep duration) were obtained from a standardized questionnaire by face-to-face interview. Physical activity was assessed using the short form of the International Physical Activity Questionnaire (IPAQ) (19), and depressive symptoms were assessed using the Patient Health Questionnaire 9 (PHQ9) (20). Nutritional status was assessed using the Malnutrition Inflammation Score (MIS) (21). Charlson Comorbidity Index (CCI) was used to assess the comorbidity risk associated with several conditions (22). We collected biochemical data including serum albumin, hemoglobin, calcium, phosphate, and parathyroid hormone (PTH) within 3 months of physical assessment. Dialysis adequacy was defined as the total fractional clearance index for urea (Kt/V) and urea reduction ratio (URR).

### Diabetes information

Access to diabetes information was based on subjects’ self-reports, and we again carefully checked the fasting plasma glucose (FPG) data through electronic medical records. According to the American Diabetes Association 2021 criteria, FPG level ≥7.0 mmol/L or 2-h plasma glucose ≥11.1 mmol/L during an oral glucose tolerance test or HbA1c ≥6.5% was considered as diabetes (23).

### Physical performance

Performance-based assessment consisted of grip strength (GS), Timed Up and Go Test (TUGT), and 4-m WS test. GS was measured using a dynamometer (GRIP-D; Takei Ltd., Niigata, Japan). Participants were allowed to exert maximum efforts twice using the dominant hand, and the average value was calculated from two attempts. TUGT assessed the seconds of standing up from a chair, walking 3 m at usual pace past a line on the floor, turning around, walking back to the chair, and then sitting down on the chair. The WS test consists of participants being timed while walking 4 m at their usual pace and they were allowed to use a gait-assistive device. Participants completed the test twice, and the mean gait speed (m/s) was calculated (17). Higher GS values, shorter TUGT, and faster WS represent better physical performance. All tests were monitored by corresponding professional physical therapists.

### MCI and cognitive function

This study adopted the MCI diagnostic criteria based on Petersen’s definitions with modifications (24): 1) memory complaints (self-reported or reported by family members or caregivers); 2) objective cognitive impairment, as assessed by the Mini-Mental State Examination (MMSE); 3) intact or only mildly impaired daily living ability, as assessed by the Instrumental Activities of Daily Living (IADL) Scale; 4) no clear dementia, as evaluated by the Chinese version of the Dementia Rating Scale (CDRS); 5) no abnormal memory impairment for age. The MMSE score ranges from 0 to 30 points, with the higher scores indicating better cognitive performance. It has been reported that the Chinese version of the MMSE indicates MCI for scores ≤17, 20, and 24 in people with the educational level of illiteracy, primary school, and middle school or higher, respectively (25). The IADL Scale includes eight items, and the score ranges from 0 to 8 points, with the higher scores indicating better daily living ability. IADL scored ≥6 indicates intact or only mildly impaired daily living ability (26). The MMSE includes a broad set of cognitive functions that measure the following: orientation (10 points), registration (3 points), attention and calculation (5 points), recall (3 points), and language (9 points).

### Statistical analysis

The baseline characteristics of participants were presented according to the classification of diabetes and MCI. Continuous variables were presented as means ± standard deviation (SD), and categorical variables were expressed as numbers and percentages. Baseline sociodemographic characteristics were analyzed using t test, Pearson’s chi-square test, and Mann–Whitney U test. The interaction effect between the component of physical performance and diabetes was tested by adding three interacted items (GS * diabetes; TUGT * diabetes; WS * diabetes) in the logistic regression analysis. Binary logistic regression analysis was used to analyze the relationship between physical performance and MCI in hemodialysis patients in the non-diabetic and diabetic groups. MCI was used as the dependent variable, each component of physical performance (GS, TUGT, WS) was used as an independent variable, and several confounding factors [age, gender, body mass index (BMI), year, widowhood, living alone, illiteracy, smoking, alcohol consumption, sleep duration, IPAQ, depression, number of medications, and CCI] were adjusted as covariates. Linear regression models were used to analyze the relationship between GS, TUGT, WS, and various cognitive functions. All of the statistical analyses were performed with the SPSS V26.0 software, and differences were defined as significant when p < 0.05.

## Results

### Participant characteristics

**Figure 1:**
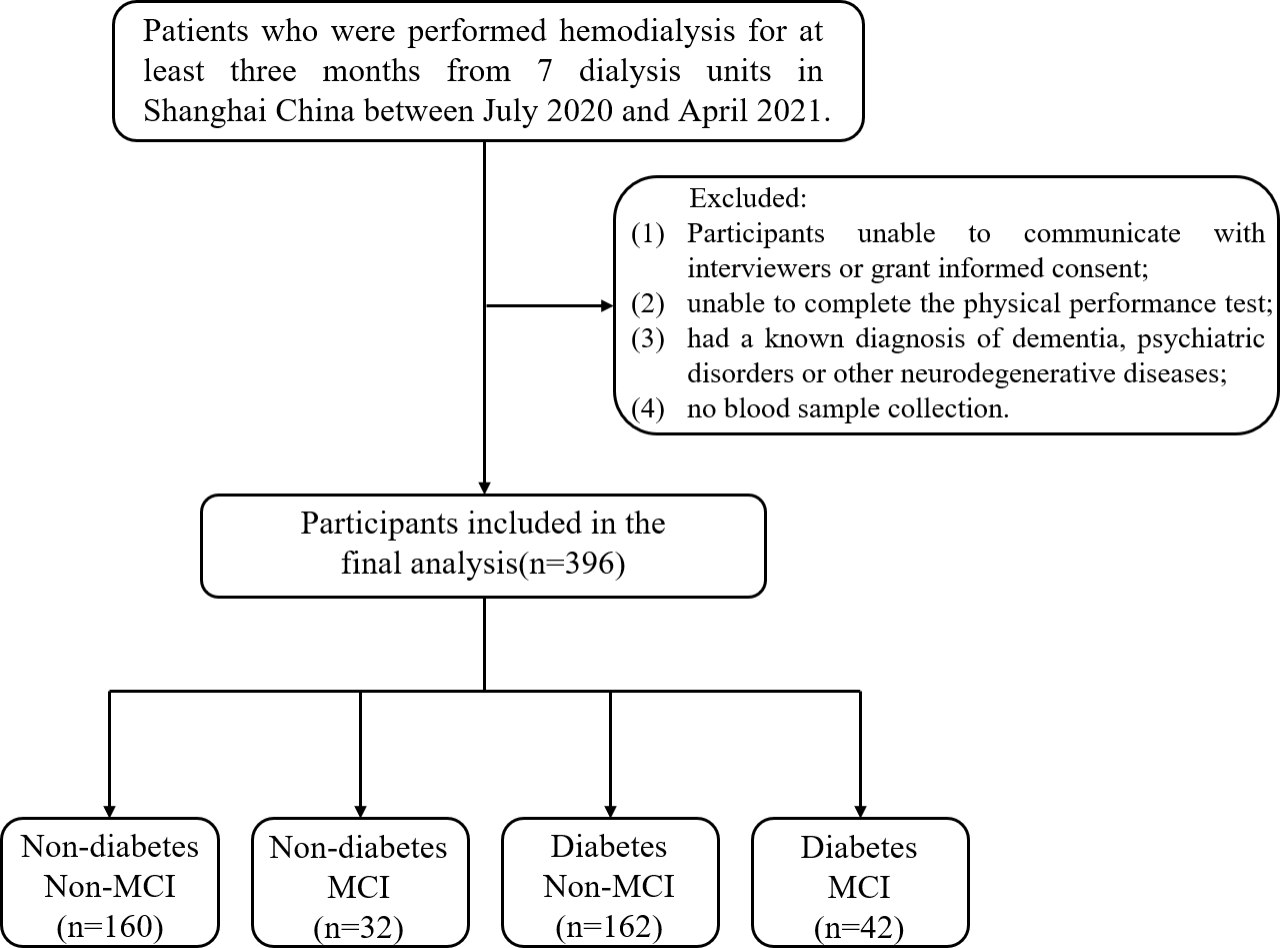
Flow diagram of the study. MCI, mild cognitive impairment.

**Figure 2:**
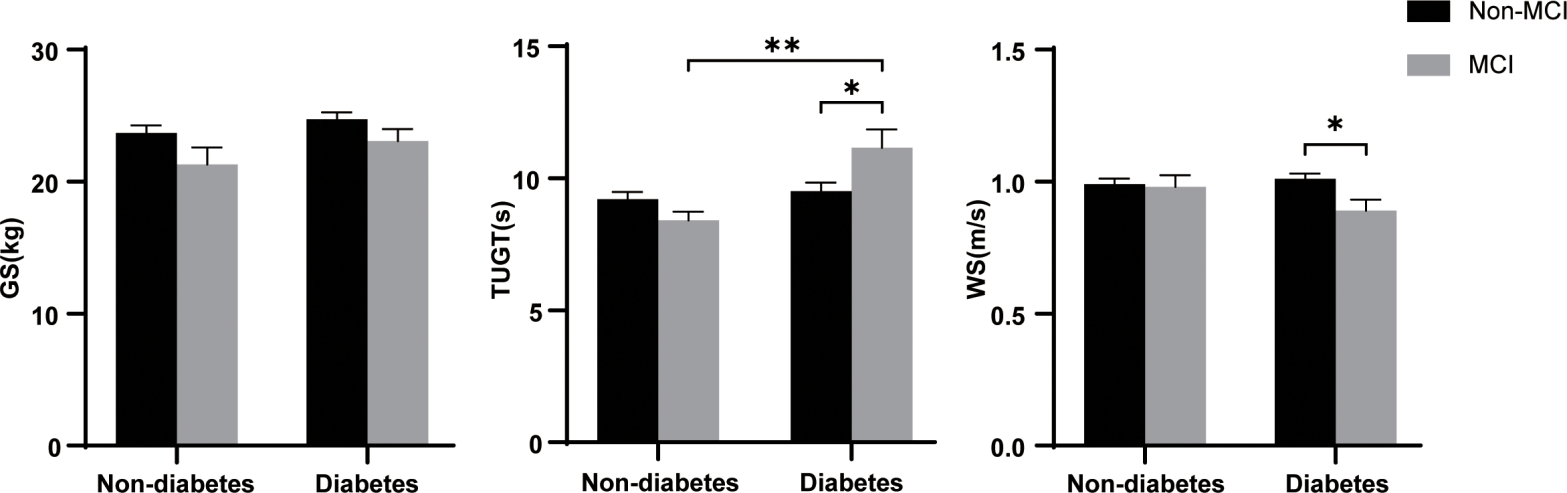
Difference of physical performance between the different groups. GS, grip strength; TUGT, Timed Up and Go Test; WS, 4-m walking speed; MCI, mild cognitive impairment. Data are presented as mean ± SD using t test. * represents p < 0.05, ** represents p < 0.01.

**Figure 1** shows the flow of hemodialysis participants with subgroups. The analysis sample consisted of 396 study participants (255 men; mean age: 68.3 ± 5.9 years). Baseline characteristics of the subjects were presented in **Table 1**. Among all participants, 204 (51.5%) reported diabetes and 74 (18.7%) had MCI. Compared to non-MCI, MCI patients with or without diabetes were prone to be widowed (p < 0.05). Compared to non-diabetes, hemodialysis patients with diabetes were prone to be men, have a shorter vintage, and have a higher CCI level. As shown in **Figure 2**, it is noteworthy that in the diabetes group, MCI patients’ physical performance (TUGT and WS) was significantly worse than that of the cognitively normal group. The TUGT of patients with diabetes was significantly longer than that of those without diabetes (p < 0.05), indicating poorer mobility. However, in the non-diabetes group, there was no significant difference in physical performance between the MCI group and the cognitively normal group.

**Table 1:** Baseline data classified by diabetes and MCI in the elderly hemodialysis patients.

Characteristics	Non-diabetes			Diabetes		
	Non-MCI (n=160)	MCI(n=32)	P	Non-MCI(n=162)	MCI(n=42)	P
Age (years)	68.58 ± 6.32	68.81 ± 6.63	0.852	67.70 ± 5.35	68.57 ± 5.82	0.359
Men (%)	97 (60.6)	13 (40.6)	0.037	118 (72.8) a	27 (64.3) b	0.276
Vintage (months)	58.6 (35.5,121.4)	46.7 (31.9,101.2)	0.226	37.1 (15.9,72.1) a	35.1 (22.2,55.2) b	0.741
BMI (kg/m^2^)	22.95 ± 3.32	22.46 ± 3.38	0.447	23.63 ± 3.48	23.97 ± 3.92	0.581
Widowed (%)	10 (6.3)	8 (25.0)	0.001	10 (6.2)	9 (21.4)	0.002
Living alone (%)	6 (3.8)	2 (6.3)	0.518	8 (4.9)	5 (11.9)	0.100
Illiterate (%)	8 (5.0)	4 (12.5)	0.110	7 (4.3)	3 (7.1)	0.450
Drinking (%)	20 (12.5)	4 (12.5)	1.000	19 (11.7)	3 (7.1)	0.393
Smoking (%)	31 (19.4)	4 (12.5)	0.358	43 (26.5)	9 (21.4)	0.498
GS (kg)	23.67 ± 7.35	21.29 ± 7.29	0.096	24.71 ± 6.89	23.08 ± 5.92	0.167
TUGT (s)	9.20 ± 3.53	8.41 ± 1.81	0.220	9.51 ± 4.24	11.15 ± 4.56 b	0.029
WS (m/s)	0.99 ± 0.27	0.98 ± 0.25	0.771	1.01 ± 0.26	0.89 ± 0.27	0.010
IPAQ (Met/week)	1882 (693,3066)	2299 (1105,4198)	0.249	1386 (693,3079) a	1473 (635,3796)	0.875
Depression (%)	65 (40.6)	17 (54.8)	0.143	63 (38.9)	11 (26.8) b	0.152
MIS score	4.09 ± 2.38	4.22 ± 2.21	0.787	4.12 ± 2.92	4.48 ± 2.68	0.471
Sleep duration (h)	8.05 ± 1.81	8.63 ± 1.87	0.106	8.35 ± 2.26	8.89 ± 1.84	0.155
MMSE score	27.02 ± 1.98	20.50 ± 3.89	<0.001	26.95 ± 2.28	21.07 ± 3.99	<0.001
CCI	3.49 ± 1.45	3.69 ± 1.42	0.491	4.38 ± 1.73 a	4.50 ± 1.61 b	0.677
**Laboratory parameters**						
Glucose (mmol/L)	5.64 ± 0.86	5.39 ± 0.76	0.132	9.82 ± 4.10 a	10.44 ± 3.88 b	0.383
Hemoglobin (g/L)	114.22 ± 13.89	113.48 ± 15.22	0.786	109.46 ± 14.00 a	112.16 ± 14.83	0.274
Albumin (g/L)	39.74 ± 3.27	39.67 ± 3.54	0.919	39.27 ± 3.24	39.41 ± 2.74	0.794
Creatinine (μmol/L)	951.46 ± 279.14	956.51 ± 176.90	0.922	931.80 ± 264.39	958.64 ± 235.90	0.554
Uric acid (μmol/L)	434.11 ± 106.91	461.45 ± 90.11	0.268	451.73 ± 81.14	446.96 ± 94.69	0.774
Calcium (mmol/L)	2.30 ± 0.26	2.25 ± 0.29	0.404	2.27 ± 0.28	2.21 ± 0.22	0.226
Phosphate (mmol/L)	1.93 ± 0.63	1.92 ± 0.52	0.945	1.87 ± 0.62	1.90 ± 0.67	0.826
PTH (pg/ml)	381.13 ± 354.86	371.62 ± 375.72	0.891	286.06 ± 238.17 a	250.83 ± 173.14	0.381
Kt/V	1.40 ± 0.31	1.44 ± 0.21	0.525	1.36 ± 0.38	1.38 ± 0.24	0.773
URR	0.68 ± 0.09	0.70 ± 0.07	0.219	0.67 ± 0.08	0.68 ± 0.06	0.632

MCI, mild cognitive impairment; BMI, body mass index; GS, grip strength; TUGT, Timed Up and Go Test; WS, walking speed; IPAQ, International Physical Activity Questionnaire; MIS, Malnutrition Inflammation Score; MMSE, Mini-Mental State Examination; CCI, Charlson Comorbidity Index; PTH, parathyroid hormone; Kt/V, fractional clearance index for urea; URR, urea reduction ratio.

Data are presented as mean ± SD or n (%).

**a** In non-MCI patients, the Diabetes group vs. the Non-Diabetes group, p < 0.05.

**b** In MCI patients, the Diabetes group vs. the Non-Diabetes group, p < 0.05.

### Associations between physical performance and MCI in the non-diabetic or diabetic hemodialysis patients

As the main findings, we investigated the association between physical performance and MCI, and the interactive effects of physical performance and diabetes were evaluated by adding the interacted items using logistic regression analysis (**Table 2**). The odds ratio (OR) of MCI for the interacted items [(TUGT) * (diabetes) and (WS) * (diabetes)] were significant (1.044, 95% confidence interval [CI] 1.002–1.087, p = 0.040; 0.905, 95% CI 0.826–0.991, p = 0.032; **Table 2**), suggesting a diabetes-dependent effect of mobility and WS. In the subgroup analysis, the crude model showed that TUGT and WS were associated with the risk of MCI in the diabetes group, and ORs (and 95% CIs) were 1.077 (1.005–1.155) and 0.181 (0.048–0.681), respectively (p < 0.05), indicating that longer TUGT was associated with a higher risk of MCI, and faster WS was associated with a lower risk of MCI, respectively. In the adjusted model (age, sex, BMI, vintage, widowed, living alone, illiteracy, smoking, drinking, sleep duration, IPAQ, depression, number of drugs, and CCI), only WS was negatively associated with MCI (p = 0.021). However, whether crude or adjusted, this association did not exist in the non-diabetes group (all p > 0.05).

**Table 2:** Logistic regression analysis of physical performance and MCI in the non-diabetic and diabetic hemodialysis patients.

Variables	Crude		Adjusted Model	
	OR (95%CI)	p	OR (95%CI)	p
**Non-Diabetes**				
GS	0.955 (0.904,1.009)	0.908	0.981 (0.902,1.071)	0.733
TUGT	0.914 (0.791,1.057)	0.225	0.863 (0.706,1.055)	0.132
WS	0.806 (0.190,3.416)	0.770	0.825 (0.113,5.731)	0.845
**Diabetes**				
GS	0.964 (0.914,1.016)	0.168	0.973 (0.906,1.048)	0.428
TUGT	1.077 (1.005,1.155)	0.036	1.084 (0.993,1.187)	0.059
WS	0.181(0.048,0.681)	0.011	0.129 (0.028,0.704)	0.021
**Interacted items**				
GS*diabetes			1.003 (0.984,1.023)	0.754
TUGT*diabetes			1.044 (1.002,1.087)	0.040
WS*diabetes			0.905 (0.826,0.991)	0.032

The interacted items were included in the above model, and the p-values for [(TUGT) * (diabetes) and (WS) * (diabetes)] were significant. Adjusted model is adjusted with age, sex, BMI, vintage, widowed, living alone, illiteracy, smoking, drinking, sleep duration, IPAQ, depression, number of drugs, and CCI.

MCI, mild cognitive impairment; BMI, body mass index; IPAQ, International Physical Activity Questionnaire; CCI, Charlson Comorbidity Index; GS, grip strength; TUGT, Timed Up and Go Test; WS, walking speed; CI, confidence interval.

### Associations between physical performance and specific cognitive functions in the hemodialysis patients

Then, we performed multivariate linear regression analysis of the association between different physical performance components and cognitive functions in the non-diabetes and diabetes hemodialysis patients (**Table 3**). In the fully adjusted model, the TUGT was negatively associated with overall cognition, attention and calculation, and language, and the WS was positively associated with overall cognition, recall, and language in the diabetes group (p < 0.05), while only WS was positively associated with attention and calculation in the non-diabetes group (p = 0.044). Whether in the non-diabetes or diabetes group, GS was not associated with any of the cognitive functions (all p > 0.05).

**Table 3:** Multivariate linear regression analysis of the association between physical performance and cognitive functions in the non-diabetic and diabetic hemodialysis patients.

Variables	Non-diabetes				Diabetes			
	Crude		Adjusted Model		Crude		Adjusted Model	
	β	p	β	p	β	p	β	p
**GS**								
MMSE score	0.070 (0.005,0.136)	0.036	0.043 (-0.032,0.114)	0.264	0.126 (0.055,0.197)	0.001	0.041 (-0.036,0.125)	0.275
Orientation	0.017 (-0.006,0.041)	0.150	0.016 (-0.015,0.040)	0.368	0.040 (0.016,0.064)	0.001	0.019 (-0.0080.050)	0.146
Registration	0.003 (-0.003,0.009)	0.288	0.001 (-0.007,0.009)	0.783	0.006 (-0.003,0.014)	0.183	0.003 (-0.007,0.015)	0.471
Attention and calculation	0.031 (0.002,0.060)	0.035	0.026 (-0.014,0.060)	0.229	0.034 (0.005,0.064)	0.023	0.005 (-0.033,0.039)	0.859
Recall	-0.005 (-0.026,0.017)	0.666	-0.005 (-0.031,0.025)	0.819	0.006 (-0.018,0.030)	0.638	0.004 (-0.025,0.036)	0.713
Language	0.024 (0.002,0.046)	0.031	0.013 (-0.014,0.033)	0.421	0.041 (0.016,0.065)	0.001	0.009 (-0.019,0.039)	0.482
**TUGT**								
MMSE score	-0.058 (-0.205,0.089)	0.439	-0.046 (-0.198,0.101)	0.527	-0.240 (-0.351,-0.130)	<0.001	-0.172 (-0.284,-0.064)	0.002
Orientation	-0.004 (-0.057,0.049)	0.887	-0.015 (-0.067,0.046)	0.718	-0.042 (-0.080,-0.004)	0.030	-0.018 (-0.055,0.025)	0.467
Registration	-0.002 (-0.015,0.011)	0.720	0.006 (-0.012,0.020)	0.622	-0.013 (-0.028,0.001)	0.064	-0.011 (-0.029,0.003)	0.114
Attention and calculation	-0.011 (-0.077,0.054)	0.731	-0.041 (-0.119,0.032)	0.257	-0.065 (-0.111,-0.019)	0.006	-0.057 (-0.108,-0.010)	0.017
Recall	0.016 (-0.031,0.064)	0.505	0.037 (-0.022,0.090)	0.236	-0.039 (-0.075,-0.002)	0.041	-0.039 (-0.077,0.006)	0.092
Language	-0.054 (-0.103,-0.006)	0.029	-0.035 (-0.080,0.016)	0.182	-0.080 (-0.118,-0.043)	<0.001	-0.049 (-0.090,-0.012)	0.014
**WS**								
MMSE score	1.861 (0.033,3.689)	0.046	1.261 (-0.497,3.026)	0.161	3.635 (1.834,5.435)	<0.001	2.827 (1.030,4.634)	0.002
Orientation	0.274 (-0.391,0.939)	0.418	0.169 (-0.499,0.832)	0.625	0.809 (0.195,1.423)	0.010	0.469 (-0.179,1.125)	0.155
Registration	0.077 (-0.086,0.239)	0.352	-0.002 (-0.192,0.187)	0.976	0.084 (-0.148,0.317)	0.474	0.034 (-0.233,0.298)	0.817
Attention and calculation	0.781 (-0.028,1.589)	0.058	0.927 (0.036,1.812)	0.044	0.738 (-0.016,1.492)	0.055	0.567 (-0.248,1.375)	0.170
Recall	0.148 (-0.447,0.743)	0.625	0.013 (-0.656,0.683)	0.962	0.950 (0.360,1.541)	0.002	1.107 (0.436,1.768)	0.001
Language	0.562 (-0.051,1.175)	0.072	0.165 (-0.400,0.735)	0.558	1.053 (0.437,1.668)	0.001	0.658 (0.017,1.306)	0.042

IPAQ, International Physical Activity Questionnaire; BMI, body mass index; CCI, Charlson Comorbidity Index; GS, grip strength; TUGT, Timed Up and Go Test; WS, walking speed; CI, confidence interval; MMSE, Mini-Mental State Examination.

Adjusted model is adjusted with age, sex, BMI, vintage, widowed, living alone, illiteracy, smoking, drinking, sleep duration, IPAQ, depression, number of drugs, and CCI.

## Discussion

The main findings of our current study showed that diabetic hemodialysis patients with MCI performed worse mobility than the non-diabetes group. Further analysis found that the interaction between mobility/WS and diabetes is significant. In hemodialysis patients with diabetes, those with MCI performed worse WS than those without MCI, whereas no association was found for patients without diabetes. Moreover, multivariate linear regression analysis showed that TUGT was negatively associated with attention and calculation and language. WS was positively associated with recall and language in diabetic hemodialysis patients.

Our previous studies have shown that physical performance was significantly different based on MCI status in Chinese older adults with an average age of 72.6 years (17). Poor health outcomes in diabetes are closely linked to physical activity and dietary patterns, which are also risk factors for CKD (27–29). Therefore, we compared physical performance in hemodialysis populations grouped by diabetes and MCI and explored the relationship between physical performance and MCI in hemodialysis patients with and without diabetes in our study. It is worth noting that we found, whether compared with MCI in the non-diabetes group or non-MCI in the diabetes group, diabetic patients with MCI have poor mobility (**Figure 2**, p < 0.05). This finding is unprecedented. Patients with CKD experience substantial loss of muscle mass, and skeletal muscle dysfunction leads to mobility limitation (30). Kestenbaum et al. (31) demonstrated a 25% reduced leg muscle mitochondrial oxidative capacity in participants with CKD and that leg muscle oxidative capacity is a significant predictor of mobility. Moreover, a history of diabetes also imparted nearly the same magnitude of reduction in mitochondrial function (31). Therefore, altered metabolic transcriptional networks and defective mitochondrial function are likely to be major mechanistic factors in the progression of CKD caused by diabetes that impairs physical function (32). In a slight departure from our previous study, we did not find an association between GS and MCI; the reason may be that our hemodialysis population cohort is relatively younger (with an average age of 68.3 years) than the elderly population cohort. Although another comparison of physical performance between the groups according diabetes showed lower physical performance in the diabetes group than the non-diabetes group of hemodialysis and peritoneal dialysis patients (33), however, so far, no research has shown that physical activity is significantly worse in the coexistence of diabetes and MCI diseases.

Furthermore, results of our study found that the prevalence of MCI in diabetic hemodialysis patients was high (20.6%). This finding was similar to the AGES–Reykjavik study (34) that showed that individuals with type 2 diabetes had poorer performance on cognitive tests than individuals without type 2 diabetes. There are several possible mechanisms for the result. First, the accumulation of glycosylation end products triggers vascular endothelial dysfunction (35), and multiple risk factors including oxidative stress, inflammation, vascular calcification, and insulin-like growth factor-1 also play roles in the development and progression of MCI (36). Second, neurodegenerative mechanisms have been proposed for the association of diabetes with MCI. The hippocampus, entorhinal formation, and frontal cortex are potential target regions in the brain that are known to have insulin receptors through which insulin-related effects may affect cognitive function (37). Diabetes may adversely affect amyloid processing and increase brain intraneuronal β-amyloid deposition (38) and tau hyperphosphorylation (39) in target regions, which is a sign of cognitive impairment. Therefore, it is reasonable to believe that diabetes in end-stage renal disease patients receiving hemodialysis may be an important risk factor for the development of MCI.

In previous studies, the relationship between physical performance and MCI in hemodialysis patients has not been fully established. In the current study, we found a significant interaction between mobility/WS and diabetes in hemodialysis patients, while the interaction between GS and diabetes was not significant. It is possible that the mechanism underlying this interaction is multifactorial. For instance, diabetes and its primary risk factors (hypertension, heart disease, and obesity) are both strongly associated with impaired mobility function and WS. Secondly, WS is associated with factors such as inflammation, neuropathy, and vascular function, which are common pathways to cognitive and physical function. However, current evidence of the association between GS and diabetes is controversial, and a study has shown no significant association between them (40). In addition, WS in physical performance was negatively associated with MCI in diabetic hemodialysis patients; however, no significant association was found in the non-diabetic group. The possible reasons are as follows: for patients with diabetes, they are significantly associated with poor physical performance, the mean TUGT was also longer in the diabetes group than that in the non-diabetes group (11.15 vs. 8.41, p < 0.05), and the mean WS was slower in the diabetes group than that in the non-diabetes group in our study (0.89 vs. 0.98) but did not show a significant difference probably due to the small sample size. Moreover, a study has shown that hyperglycemia is associated with the development of frailty and incident mobility limitations, potentially mediated by loss of muscle (41). This is also consistent with previous findings supporting a role of specific cardiovascular risk factor contributors in the association between physical performance and cognitive decline (42). Therefore, it is reasonable to believe that poor physical performance due to diabetes may be an important risk factor for the development of MCI. This finding takes our pinpointing of amenable factors for MCI a step further, and physical performance interventions in more precise populations may be useful for early prevention and control of MCI progression.

Moreover, we found that TUGT was negatively associated with not only global cognitive function but also several specific functions, including attention and calculation and language. WS was positively associated with recall and language even after adjusting for potential confounding factors. Recent studies have revealed a strong relationship between gait and executive functions in healthy and pathological aging. The main negative correlations were found between time of TUGT and total score (r = –0.476) and language domain (r = –0.448) in the MCI group (43). McGough etal. (44) found that slow gait was associated with registration, attention and calculation, and executive performance. This is consistent with our findings that showed that WS was positively associated with attention and calculation (p = 0.044) in the non-diabetes group, and TUGT was negatively associated with language in the diabetes group. The following clinically relevant links can explain our results: cognitive function is related to the dorsolateral frontal cortex and hippocampus, which affect the executive function, attention and calculation, and recall of individuals. On the flip side, gait decline increases the risk of cognitive decline and dementia, and poor mobility outcomes were reliably associated with reduced gray and white matter volume (45). At present, although many consistent studies showed the relationship between physical activity and cognitive functions, there are still some inconsistent results (16, 44). Future studies should focus on the different cognition changes in the weak physical population, and more well-designed cohort studies need to be carried out to verify the relationship between physical performance and different cognitive functions. Generally, our finding gives us some inspiration on how to manage physical activity and interfere with MCI in hemodialysis patients, especially those with diabetes.

The strengths of our study included the following: It is the first multicenter study to examine the relationship between physical performance and MCI among hemodialysis patients across different diabetic states. Secondly, the study assessed the association between physical performance and multiple cognitive functions in hemodialysis patients with and without diabetes. Furthermore, most recognized confounders were taken into account in regression models to analyze the independent association of physical performance and MCI in this study. However, some limitations also exist. First, all participants in the present study come from one city, which means that this study has a certain degree of regional limitation. Second, this study is based on a cross-sectional design, so it is not possible to determine causal relationships. To clarify this issue, a further longitudinal study with a large sample size is needed to explore the new onset risk of MCI in the hemodialysis population with diabetes.

## Conclusion

In this study, we found that physical performance was associated with MCI in diabetic hemodialysis patients rather than the non-diabetes group. Further analysis showed the relationship between physical performance and specific cognitive functions. This study provides some key considerations for physicians that poor mobility and WS in diabetic hemodialysis patients are more associated with MCI. Further research is required to confirm the direction of causality.

## Data availability statement

The raw data supporting the conclusions of this article will be made available by the authors, without undue reservation.

## Ethics statement

The study was reviewed and approved by the Ethics Committee of Shanghai University of Medicine and Health Sciences and the methods were carried out in accordance with the principles of the Declaration of Helsinki. The patients/participants provided their written informed consent to participate in this study.

## Author contributions

YZ, PS and CZ contributed equally to this work and should be considered as the co-first authors. XC, PS and YZ conceived the concept and design of the study. WD, JN, JZ, XS, LMZ, CY and JX provided the study materials or patients. CZ, LYZ and YZ collected and assembled the data. PS, CF, PH and HZ analyzed and interpreted the data. YZ, PS and CZ drafted the article or revising it critically for important intellectual content. QG provided administrative support. All authors contributed to the article and approved the submitted version.

## Funding

This work was supported by the National Natural Science Foundation of China (No. 82172552) and the Key Clinical Support Specialty Construction Project of Shanghai Hongkou District (HKZK 2020B02).

## Acknowledgments

We thank all the medical staff at the multi-center dialysis for their generous technical assistance and guidance. We also thank all the study participants for their kind participation and cooperation.

## Conflict of interest

The authors declare that the research was conducted in the absence of any commercial or financial relationships that could be construed as a potential conflict of interest.

## Publisher’s note

All claims expressed in this article are solely those of the authors and do not necessarily represent those of their affiliated organizations, or those of the publisher, the editors and the reviewers. Any product that may be evaluated in this article, or claim that may be made by its manufacturer, is not guaranteed or endorsed by the publisher.
